# Psychometric evaluation of the Spanish version of the therapeutic communication scale in nursing students

**DOI:** 10.1371/journal.pone.0332615

**Published:** 2025-09-15

**Authors:** Jaime Carballedo-Pulido, Bárbara Hurtado-Pardos, Juan Roldán-Merino, Marta Berenguer-Poblet, Manuel Tomas-Jimenez, Montse Lamoglia-Puig, Emma Gómez Fernández, Marta Domínguez-del-Campo, Carla Otero-Arús, Mariona Farres-Tarafa, Susana Santos-Ruiz

**Affiliations:** 1 Campus Docent Sant Joan de Deu, Universitat Vic-Universitat Central de Catalunya (UVIC-UCC), C/ Sant Benito Menni, Sant Boi de Llobregat, Barcelona, Spain; 2 Mental Health, Psychosocial and Complex Nursing Care Research Group-2021 SGR, Spain; 3 Nursing Department, Campus Terres de l’Ebre, Universitat Rovira i Virgili, Tortosa, Spain; 4 Research Group on Advanced Nursing (CARING)-161, Universitat Rovira I Virgili, Tarragona, Spain; 5 Patient Safety Research Group, Parc Sanitari Sant Joan de Déu, Sant Boi de Llobregat, Spain; 6 Parc Sanitari Sant Joan de Déu, Sant Boi de Llobregat, Spain; 7 Department of Fundamental and Clinical Care Nursing, Hospitalet del Llobregat, Universitat de Barcelona, Campus de Bellvitge, Barcelona, Spain; 8 Parc Taulí Hospital Universitari. Institut d’Investigació i Innovació Parc Taulí (I3PT-CERCA). Universitat Autònoma de Barcelona. Sabadell, Spain; 9 Grup d’Investigació DAFNiS (Dolor, Activitat Física, Nutrició i Salut), Sant Boi de Llobregat, Spain; School of Nursing Sao Joao de Deus, Evora University, PORTUGAL

## Abstract

**Background:**

Therapeutic communication is a core component of person-centered nursing care. Despite its clinical relevance, few validated instruments exist in Spanish that objectively assess this competence in undergraduate nursing students.

**Objective:**

This study aimed to adapt and validate the Spanish version of the Therapeutic Communication Scale in Nursing Students (TCS-NS), originally developed by Han et al. (2024), and to examine its psychometric properties in a Spanish-speaking context.

**Methods:**

A psychometric study was conducted in two phases: (1) translation and cross-cultural adaptation following international guidelines, including expert panel review and pilot testing; and (2) psychometric evaluation with a sample of 468 nursing students. Construct validity was assessed using confirmatory factor analysis (CFA), and reliability was examined via Cronbach’s alpha, McDonald’s omega, composite reliability, and test-retest stability (ICC).

**Results:**

The CFA supported the two-factor structure of the TCS-NS and indicated good model fit (CFI = .963; TLI = .954; RMSEA = .053; SRMR = .044). Internal consistency was adequate for the total scale (α = .880; ω = .884), and composite reliability was high (.936). The intraclass correlation coefficient for the total score was.696 (95% CI:.605–.766).

**Conclusions:**

The Spanish version of the TCS-NS is a valid and reliable instrument for assessing therapeutic communication skills in nursing students. Its use may contribute to the objective evaluation of communication competence in educational interventions and research.

## Introduction

Therapeutic communication is understood as a deliberate interpersonal process in which the nurse uses verbal and non-verbal strategies to explore, clarify, and address the emotional, cognitive, and spiritual needs of the patient. Its foundational importance can be traced back to the work of Hildegard Peplau, who emphasized therapeutic interpersonal relationships as the core of professional nursing practice, conceiving them as intentional, dynamic, and reciprocal interventions aimed at supporting the growth of both the patient and the nurse. This process is particularly relevant in the identification phase of her theory [1,2]. Decades later, contemporary approaches continue to affirm that therapeutic communication is the foundation of person-centered care and a key transversal component of advanced nursing practice [3,4].

The clinical relevance of therapeutic communication has been firmly established through multiple meta-analyses and systematic reviews. The review by Haskard Zolnierek and DiMatteo showed that poor communication increases the risk of non-adherence by 19%, while targeted communication training improves adherence by up to 62% [5]. Additionally, the pathways model proposed by Street et al. (2009) suggests that effective communication between healthcare professionals and patients can enhance health outcomes through mechanisms such as increased treatment adherence, better emotional coping, and positive physiological changes [6]. Recent studies confirm that nurses’ communication competence is directly associated with improved quality of life, reduced pain, and higher patient satisfaction [7,8]. Conversely, the absence of structured communication within healthcare teams also has serious consequences: according to the Joint Commission, nearly one-fourth of sentinel events reported in 2023 were related to clinical communication failures [9].

Several studies have identified significant challenges in the development of these skills during undergraduate training. A recent systematic review based on constructivist learning environments highlighted that educational interventions incorporating cognitive tools and collaborative strategies, such as coaching, are effective in improving therapeutic communication among nursing students [10].

Moreover, a concept analysis using the Walker and Avant method highlighted the importance of therapeutic communication as a key clinical competency, emphasizing the need for both theoretical and practical education that includes constructive feedback from educators [11]. These findings suggest that the integration of structured, student-centered educational approaches can strengthen therapeutic communication skills, thereby contributing to more person-centered care and improved health outcomes.

The World Health Organization identifies therapeutic communication as a cornerstone of the framework for people-centred and integrated health services, recognizing it as essential for achieving safe and equitable outcomes in healthcare systems [12]. In the educational domain, the American Association of Colleges of Nursing includes communication as a core competency domain for both undergraduate and graduate nursing graduates, requiring its demonstration in clinical, community, and interprofessional contexts [13].

However, evidence points to persistent deficits in therapeutic communication skills among nursing students and newly graduated professionals [14]. Members of Generation Z, shaped by digital socialization and a culture of immediacy, often experience anxiety and face-to-face communication difficulties during clinical placements [15,16]. These challenges have been further exacerbated in the aftermath of the COVID-19 pandemic, with students reporting increased stress, greater language-related barriers, and limitations in establishing empathetic connections with patients—particularly older adults [15,17].

The persistence of these deficits may be partly attributed to the limitations of traditional teaching methods in effectively addressing the needs of new generations and the emerging challenges in the post-pandemic context. The inherent complexity of therapeutic communication—which requires not only theoretical knowledge but also the development of advanced practical and reflective skills—may be another contributing factor.

To address this gap, nursing education programs have implemented various methodologies such as simulation with actors (standardized patients), which enables communication training in a psychologically safe environment, virtual reality, and constructivist approaches incorporating tools such as case-based learning, cognitive instruments, and coaching. These strategies have shown particular effectiveness in promoting therapeutic communication competence [8].

Interventions involving standardized patients improve students’ self-confidence and perceived communication competence [18]. Virtual reality in nursing education provides an immersive environment that supports learning through trial and error without compromising patient safety and enhances the acquisition of clinical and communication competencies [19].

However, systematic reviews have identified three main barriers: the heterogeneity of study designs, which hinders comparison across studies; the deployment of standardized patients and advanced technology, which significantly increases costs; and the lack of evidence confirming that skills acquired in simulated settings are transferred to real clinical care [20–22].

In this context, the objective assessment of therapeutic communication is essential for designing and comparing effective educational interventions. While instruments such as the Global Interprofessional Therapeutic Communication Scale (GITCS) and the questionnaire developed by Ghiyasvandian et al. exist [23–25], they present limitations regarding their development context and conceptual focus. Specifically, in Spanish, available instruments are limited to those measuring attitudes or self-perception, which constrains the behavioral assessment of therapeutic communication in undergraduate nursing students [26–29].

In 2024, Han, Yoo, and Kang introduced the Therapeutic Communication Scale in Nursing Students (TCS NS) [10], specifically designed to assess behavioral competence in therapeutic communication among undergraduate nursing students. Its factorial structure, aligned with person-centered practice, and its sensitivity to educational changes make it a strong candidate for inclusion in Spanish-speaking training programs [10].

However, the practical utility of any measurement instrument critically depends on its psychometric properties. The specialized literature recommends rigorous processes of cross-cultural adaptation [30,31], and the COSMIN initiative provides standards for assessing the methodological quality of psychometric property studies [32,33]. These normative frameworks emphasize the importance of ensuring content validity, internal structure, reliability, and responsiveness of the scale prior to its implementation in educational or research settings.

Therefore, the primary objective of this study is to conduct the Spanish adaptation and psychometric validation of the Therapeutic Communication Scale in a sample of nursing students. This adapted version is expected to provide a valid and reliable tool for the objective assessment of therapeutic communication skills within Spanish-speaking educational contexts, thereby contributing to improving communication competence training and, ultimately, to enhancing person-centered care.

## Methods

### Study design

A psychometric study was conducted to carry out the cross-cultural adaptation into Spanish and the validity analysis of the Therapeutic Communication Scale in Nursing Students *Students* [10]. The study was conducted in two phases. In the first phase, the scale was translated and culturally adapted into Spanish. In the second phase, the psychometric properties of the Spanish version of the instrument were analyzed.

### Study setting and sample

The psychometric properties of the scale were analyzed in a sample of undergraduate nursing students from Campus Docent Sant Joan de Déu, Universitat de Vic – Universitat Central de Catalunya. Data collection was conducted between April 15 and May 20, 2025.

A non-probability convenience sampling method was used: all students present in class during the data collection period who met the inclusion criterion of having completed at least one clinical placement in a hospital or community care setting and were enrolled in the second, third, or fourth academic year were invited to participate. The only exclusion criterion was being absent from class on the day the questionnaire was administered.

For data collection, a structured form was designed consisting of two sections. The first section gathered sociodemographic data, and the second included the Spanish version of the Therapeutic Communication Scale in Nursing Students, the instrument under validation in this study.

The sample size determination was based, first, on the guidelines proposed by Comrey and Lee (1992), who offer a benchmark scale for evaluating sample adequacy in factor analysis: 100 cases are considered poor, 200 fair, 300 good, 500 very good, and 1,000 excellent [34]. Based on this framework, a minimum target of 500 students from different academic years was set, ensuring a “very good” sample size. In addition, a methodological criterion was applied: confirmatory factor analysis requires at least 150 participants to generate stable estimates [35,36]. The combination of both approaches supports the adopted threshold and reinforces the robustness of the resulting sample.

### Variables and source of information

Sociodemographic and academic variables were collected from the participating students: age, gender, academic year, enrollment shift, employment status (whether they were working at the time of the study and, if so, whether the activity was in the healthcare sector), and motivation for choosing the nursing degree (voluntary choice vs. recommendation by others). In addition, the Spanish version of the Therapeutic Communication Scale in Nursing Students, the instrument under validation in this study, was administered.

The final Spanish version of the TCS consists of 15 items clustered into two latent factors.

Factor 1 – Relation Building (9 items) captures empathy/expressing concern (item 1), empathy/behavioral cues (item 2), patient-centered environment/support (item 3), reaffirmation (item 4), comfort/adequate voice (item 5), giving information/feedback (item 6), noticing (item 7), accepting (item 8), and setting limitations (item 9).

Factor 2 – Problem Solving (6 items) addresses caring/professional attitude (item 10), factual information exchange (item 11), clarification (item 12), listening (item 13), truth/honesty (item 14), and giving enough time (item 15).

Each item is rated on a 4-point Likert scale: 1 = strongly disagree, 2 = disagree, 3 = agree, 4 = strongly agree. Total scores range from 15 to 60, with higher scores indicating higher levels of therapeutic communication skills. Subscale scores are obtained by summing the corresponding items within each factor.

The distribution of items across factors, along with their characteristics, is presented in Table 1.

**Table 1:** Item distribution by factor and minimum/maximum scores in the original version of the Therapeutic Communication Scale in Nursing Students, developed by Han et al.

Factors	Items	Minimum	Maximum
Factor 1: Relation building	Items from 1 to 9	9	36
Factor 2: Problem solving	Items from 10 to 15	6	24
Scale Total	Items from 1 to 15	15	60

### Procedure

The translation and back-translation process of the scale was conducted in accordance with the recommendations of the *Standards for Educational and Psychological Testing* and the International Test Commission. First, permission was requested and obtained from the original authors to culturally adapt the scale to the Spanish context. The original English version was then independently translated into Spanish by two sworn translators with no prior experience with the scale and no knowledge of the study objectives.

Next, a panel of experts was assembled, consisting of seven nursing professionals: two with clinical practice experience, three with academic teaching profiles, and two with specialized training in psychometrics. The aim of the panel was to review the semantic equivalence between the original and translated versions. The meaning and clarity of each item were compared across both versions to ensure consistent conceptual interpretation in both languages. All items were retained in the preliminary Spanish version, and semantic equivalence was unanimously agreed upon by all panel members.

Additionally, the relevance of each item to the Spanish cultural and academic context was assessed using a 4-point Likert scale (1 = not relevant, 2 = slightly relevant, 3 = quite relevant, 4 = highly relevant). A minimum content validity index (CVI) of 0.80 was established as the acceptance threshold. This index was calculated by dividing the number of expert ratings of “quite relevant” or “highly relevant” by the total number of experts. All items achieved CVI values between 0.83 and 1.00, confirming their adequacy.

Finally, the consensus Spanish version was back-translated into English by two independent translators. The back-translation was reviewed by the research team and compared with the original version, and no discrepancies were identified that warranted modifications.

As the final phase of the adaptation process, a pilot study was conducted with 20 undergraduate nursing students from different academic years to assess the clarity, comprehensibility, and linguistic appropriateness of the items, as well as the time required to complete the questionnaire. After a feedback session with participants, no modifications to the content or format of the scale were deemed necessary. Table 2 presents the semantic equivalence of the items between the original English version and the Spanish adaptation.

**Table 2:** Semantic equivalence of items between the original English version and the Spanish adaptation of the Therapeutic Communication Scale in Nursing Students.

Item	English	Spanish
Item 1	I verbalize about clients’ situation or how they feel.	Verbalizo la situación de los usuarios o como se sienten
Item 2	I express how I understand the client by gestures and eye contact.	Expreso cuanto entiendo al usuario mediante gestos y contacto visual
Item 3	I build a comfortable atmosphere for conversation.	Creo una atmósfera confortable para la conversación
Item 4	I recheck clients’ words and behaviors when they are not clear.	Verifico las palabras y conductas de los usuarios cuando no son claras
Item 5	I use an appropriate tone and volume when I talk.	Utilizo un tono y volumen apropiados al hablar
Item 6	I give information easy enough for the client to understand.	Proporciono información lo suficientemente sencilla para que el usuario la entienda
Item 7	I notice changes in body language, facial expressions, and emotions even if the patient does not speak.	Percibo cambios en el lenguaje corporal, las expresiones faciales y las emociones, incluso si el usuario no habla
Item 8	I meet clients without prejudice.	Atiendo a los usuarios sin prejuicios
Item 9	I explain I can’t approve unreasonable demands of the client.	Explico que no puedo aceptar demandas poco razonables del usuario
Item 10	I don’t give advice or recommendations without clients’ approval.	No doy recomendaciones ni consejos sin la aprobación de los usuarios
Item 11	I don’t assume what the client will say.	No presupongo lo que el usuario va a decir
Item 12	I only ask the patient one question at a time.	Hago al usuario solo una pregunta a la vez
Item 13	I listen to the patient’s speech until the end without interrupting or blocking.	Escucho el discurso del usuario hasta que termina sin interrumpirlo ni bloquearlo
Item 14	I don’t reduce or exaggerate the clients’ problem.	Ni minimizo ni exagero los problemas de los usuarios
Item 15	I give enough time for the client to organize their thoughts.	Doy el tiempo suficiente para que el usuario organice sus pensamientos

The administration of the questionnaire was scheduled by assigning a specific date for each academic year and enrollment shift, ensuring that all students had the opportunity to participate. Prior to completing the instrument, students received a verbal explanation of the study’s objectives and were invited to participate on a fully voluntary basis. Data collection was carried out using the REDCap platform, which allowed for the secure and structured administration of the questionnaire and the storage of responses.

### Ethical considerations

The study was approved by the Ethics and Research Committee of the Campus Docent Sant Joan de Déu (approval code: PR2/25). All participants were informed about the objectives of the study and were assured that the data collected would remain anonymous. On the day of completing the scale, all participants were verbally informed about the study and were asked to provide written informed consent prior to participation

### Data analysis

The statistical package SPSS version 29.0 for Windows (SPSS Inc., Chicago, IL, USA) was used for data analysis, and EQS version 6.3 was used for confirmatory factor analysis (CFA).

### Construct validity

To assess the structural validity of the Spanish version of the Therapeutic Communication Scale, a confirmatory factor analysis (CFA) was performed using the maximum likelihood (ML) estimation method. This approach estimates model parameters under the assumption of multivariate normality and is widely employed in psychometric research due to its efficiency and comparability. Although the items in the scale follow an ordinal response format with five categories, several authors have noted that, in large samples (n ≥ 300) and when a sufficient number of response categories is present, ML estimation remains appropriate even with moderate deviations from normality [37,38]. To confirm the robustness of the estimation, results were also compared with those obtained via least squares (LS) estimation, yielding very similar psychometric outcomes. Based on existing literature and the available sample size (n = 468), ML was selected as the primary estimation strategy.

To assess the overall model fit, the following indices were used: χ²/df, Comparative Fit Index (CFI), Tucker–Lewis Index (TLI), Goodness-of-Fit Index (GFI), Adjusted Goodness-of-Fit Index (AGFI), Root Mean Square Error of Approximation (RMSEA, with 90% CI), and Standardized Root Mean Square Residual (SRMR). The following thresholds were considered indicative of good model fit: CFI and TLI ≥ .95, χ²/df between 2 and 5, RMSEA ≤ .06, SRMR ≤ .08, and GFI and AGFI ≥ .90 [39–41].

### Reliability

Internal consistency of the instrument was assessed using Cronbach’s alpha and McDonald’s omega coefficients, calculated for both the total score and each specific factor. Additionally, composite reliability was estimated to provide a more comprehensive view of the instrument’s quality. To evaluate temporal stability, a test–retest analysis was conducted with a 10-day interval using the intraclass correlation coefficient (ICC) in a subsample of 225 nursing students [42].

### Results

**Table 3:** Sociodemographic characteristics of nursing students participating in the validation of the Spanish version of the Therapeutic Communication Scale. (n = 468).

Age, mean (SD)	22.8 (4.6)	
**Sex**	n	%
Women	389	83,1
Men	76	16,2
Non binary	1	0.2
No response	2	0.4
**Year academic**		
Second	178	38.0
Third	184	39.3
Fourth	106	22.6
**Work**		
Yes	286	61.1
No	182	38.9
**Health care field**		
Yes	148	51.7
No	138	48.3
**Motivation for choosing the degree program**		
Voluntary	442	94.4
Recommended by family or peers	26	5.6

### Participant characteristics

The sample was predominantly composed of women (83.1%), with a mean age of 22.8 years (SD = 4.6). Most participants were enrolled in either the second (38.0%) or third year (39.3%) of the nursing degree program, and 61.1% reported being employed at the time of data collection. Those who were working were asked whether their job was in the healthcare sector; 51.7% responded affirmatively. Finally, 94.4% stated that the decision to pursue a nursing degree had been voluntary. Full sociodemographic characteristics of the sample are presented in Table 3.

### Construct validity

#### Confirmatory factor analysis (CFA).

**Fig 1:**
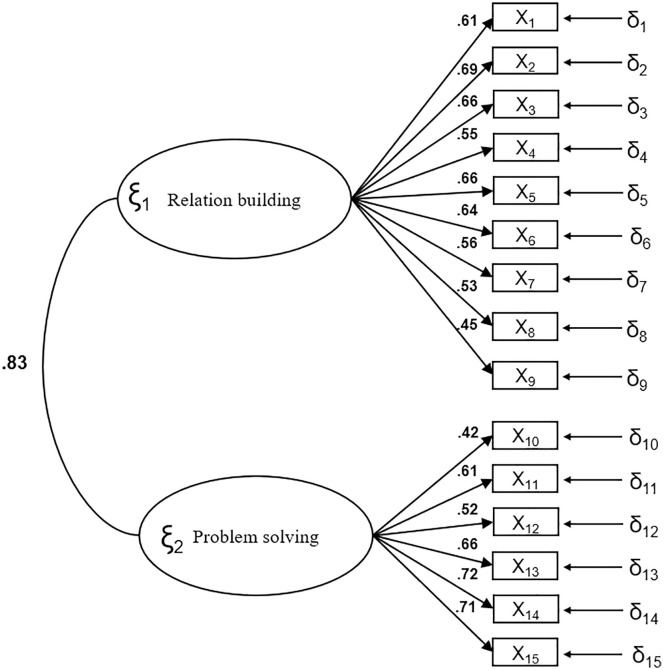
Standardized model parameters.

A confirmatory factor analysis was conducted to assess the two-factor structure of the Spanish version of the Therapeutic Communication Scale in Nursing Students. The model fit results are presented in Table 4, showing satisfactory values across all indices. Fig 1 illustrates the standardized solution of the model, which confirmed the expected factorial structure with two latent variables: “Relation Building” (Items 1–9) and “Problem Solving” (Items 10–15). All items loaded significantly onto their respective factors, and the correlation between the two latent constructs was.83, indicating a strong association while preserving conceptual distinction between the dimensions.

**Table 4:** Goodness-of-fit indices of the confirmatory model of the Spanish version of the Therapeutic Communication Scale in Nursing Students.

INDEX	VALUE
CFI	0.952
TLI	0.943
GFI	0.947
AGFI	0.920
SRMR	0.042
RMSEA	0.050 (CI. 0.040–0.059)
Cronbach’s alpha	0.880
Goodness of fit test	X^2^ = 191,601; df = 89; p < 0,0001
Reason for fit	X^2^/df = 2,15

CFI: Comparative Fit Index. TLI: Tucker–Lewis Index. GFI: Goodness-of-Fit Index. AGFI: Adjusted Goodness-of-Fit Index. SRMR: Standardized Root Mean Square Residual. Root Mean Standard Error of Approximation.

### Reliability

The instrument demonstrated adequate internal consistency, with a Cronbach’s alpha coefficient of 0.880 for the total score and values above 0.77 for the individual factors. The omega coefficient (ω) for the total scale was 0.884, and the composite reliability yielded a global value of 0.936. Regarding test-retest reliability, the intraclass correlation coefficient (ICC) for the total score was 0.696 (95% CI: 0.605–0.766). Full results are presented in Table 5.

**Table 5:** Cronbach’s alpha coefficient, omega coefficient and ICC test–retest (*n* = 225).

Factors	Cronbach’s alpha	Omega (ω)	Composite reliability	ICC (CI 95%)
Factor 1: Relation building	0.830	0.835	0.898	0.621 (0.508 - 0.709)
Factor 2: Problem solving	0.778	0.787	0.853	0.697 (0.606 - 0.767)
Total Score	0.880	0.884	0.936	0.696 (0.605 - 0.766)

CI, confidence interval; ICC, intraclass correlation coefficient.

Additional information on the characteristics of each item, including descriptive statistics and factor loadings, has been provided in the Supporting Information file.

## Discussion

This study provides robust evidence on the psychometric properties of the Spanish version of the Therapeutic Communication Scale in Nursing Students, confirming both its structural validity and reliability as a tool for assessing behavioral competence in therapeutic communication.

The confirmatory factor analysis (CFA) supported the original bifactorial structure proposed by Han et al. (2024), successfully replicating the distribution of items across the “Relationship Building” and “Problem Solving” dimensions. All global fit indices fell within ranges considered acceptable or excellent according to established methodological standards [39–41], supporting the theoretical coherence of the model within nursing education settings in Spain.

Although the items followed an ordinal response format, the maximum likelihood (ML) method was selected for parameter estimation based on prior evidence supporting its appropriateness in large samples with five or more response categories, even under moderate violations of multivariate normality [37,38]. In this study, the sample size (n = 468) and the distributional properties of the items supported the use of ML. Furthermore, the similarity of results obtained using least squares (LS) estimation reinforces the stability and robustness of the model.

The incremental fit indices (CFI, TLI) exceeded the.95 threshold, which is typically interpreted as evidence of good model fit [39]. Additionally, the error-based indices, including RMSEA and SRMR, remained below the recommended cutoff values (.06 and.08, respectively), indicating minimal discrepancy between the observed and estimated covariance matrices [40,41] its value fell within the acceptable range (between 2 and 5), further supporting the global adequacy of the model.

The reliability analysis yielded satisfactory results across all indicators. Both Cronbach’s alpha and McDonald’s omega exceeded.77 for the overall scale and its subdimensions, reflecting strong internal consistency. Of particular note, McDonald’s omega is considered a more accurate estimate of reliability in multidimensional instruments because it does not require the assumption of equal factor loadings [43,44]. Composite reliability for the total scale was.936, suggesting a high degree of measurement precision [40]. Furthermore, test–retest reliability assessed through the intraclass correlation coefficient (ICC) was.696, a level deemed acceptable for evaluating competencies that are context-sensitive and may be influenced by situational variability [45,46].

The results of this study support the utility of the Therapeutic Communication Scale in Nursing Students as a psychometrically robust instrument for assessing students’ self-perceived competence in therapeutic communication. By encompassing behavioral, cognitive, and relational dimensions that are fundamental to nursing practice, the scale offers a diagnostic framework to evaluate communication skills expected to develop during undergraduate training. Its application may be valuable in both academic and clinical contexts. Nevertheless, as a self-report measure, the scale captures subjective perceptions and does not necessarily reflect actual performance. Therefore, it is recommended that its use be complemented with direct or observational assessment strategies, particularly when the aim is to evaluate learning outcomes or applied skills in care settings.

### Study strengths and limitations

One of the main strengths of this study is the rigorous methodological process employed for the cross-cultural adaptation and validation of the Therapeutic Communication Scale in Nursing Students. Internationally recognized guidelines were followed, including forward and back translation, expert panel review, pilot testing, and psychometric evaluation in a large and diverse sample. In addition, robust statistical techniques were applied, such as confirmatory factor analysis and multiple reliability indicators (Cronbach’s alpha, McDonald’s omega, composite reliability, and intraclass correlation coefficient), reinforcing the credibility of the findings. The scale assesses behavioral components of therapeutic communication, addressing a gap in existing Spanish-language instruments, which tend to focus on attitudes or perceived self-efficacy.

Despite these strengths, several limitations should be acknowledged. First, the sample was drawn from a single academic institution in Spain using a non-probabilistic convenience sampling approach. Although the sample size was adequate for the statistical analyses, the limited representation of other universities and regions may restrict the generalizability of the results. Future research should include participants from multiple educational settings and geographic regions to enhance external validity.

Second, the use of a self-report instrument carries inherent limitations, such as social desirability bias and the tendency to respond in line with perceived expectations. While self-report tools are commonly used in psychometric research, their results do not necessarily reflect actual communicative behavior in clinical contexts. The absence of an observational or performance-based reference criterion is a notable limitation, especially given the applied nature of therapeutic communication.

Third, although test–retest reliability was assessed with a 10-day interval, this short period may have overlapped with key reflective moments during students’ clinical placements. For instance, feedback or evaluation sessions with clinical mentors may have influenced their self-assessment of communication skills, potentially altering responses in the second administration. This could have introduced variability unrelated to the instrument’s temporal stability, reinforcing the need for longer and contextually stable intervals in repeated measures.

Finally, the study did not address convergent, discriminant, or criterion validity. These dimensions are essential to determine whether the scale correlates appropriately with related constructs, differentiates between varying levels of competence, or predicts meaningful outcomes. Future studies should incorporate complementary instruments and external criteria to address this limitation.

Moreover, although the present study focused exclusively on the Spanish version of the Therapeutic Communication Scale in Nursing Students, the methodological process applied here provides a solid foundation for future cross-cultural adaptations. Given that the underlying constructs of therapeutic communication, such as relationship building and problem solving, are relevant across health care contexts, the scale may be suitable for translation into other languages. Nevertheless, the psychometric properties observed in this study cannot be assumed to generalize automatically. Any future adaptation should follow rigorous cross-cultural validation procedures, including semantic, conceptual, and metric equivalence testing, to ensure the instrument’s reliability and validity in non-Spanish-speaking populations.

In addition, it is important to encourage future validation studies across diverse academic settings to test the generalizability of the scale in different institutional and cultural contexts. Such studies should not only replicate the factorial structure and reliability estimates but also incorporate convergent and criterion validity measures by comparing the scale with related constructs and external performance indicators. This would provide stronger evidence of its construct validity and extend its applicability as an assessment tool for nursing education internationally.

## Conclusions

This study provides robust evidence supporting the validity and reliability of the Spanish version of the Therapeutic Communication Scale in Nursing Students (TCS-NS) as a psychometrically sound instrument for assessing behavioral competencies in therapeutic communication. The confirmatory factor analysis confirmed the two-factor structure of the original version, with satisfactory fit indices and internal consistency across both dimensions. Additionally, the instrument demonstrated adequate temporal stability and high composite reliability, strengthening its applicability for educational and research purposes.

The availability of a culturally adapted and psychometrically validated instrument in Spanish addresses a critical gap in nursing education and enables the systematic evaluation of communication skills beyond self-efficacy perceptions or attitudinal constructs. The use of this scale may help educators monitor learning outcomes, tailor communication training programs, and ultimately improve the quality of patient-centered care.

Nevertheless, self-reported measures should be interpreted with caution and ideally complemented with observational assessments or performance-based evaluations to provide a more comprehensive picture of communicative competence. Future research should expand the validation of the scale across diverse populations and explore its sensitivity to change in response to targeted educational interventions.

## Supporting information

S1. Table Characteristics of each item, including descriptive statistics and factor loadings. This file contains three tables (Table S1.1, Table S1.2, Table S1.3).
(DOCX)

S1 Dataset Data underlying the analyses. (XLSX)
