# Supplementary material for: Psychometric evaluation of the Spanish version of the therapeutic communication scale in nursing students
Source: PLoS One. 2025 Sep 15;20(9):e0332615. doi: 10.1371/journal.pone.0332615 (PMC12435668; doi:10.1371/journal.pone.0332615)
Supplement: S1. Table — This file contains three tables (Table S1.1, Table S1.2, Table S1.3). (DOCX) [file pone.0332615.s001.docx]

| **S1.1 Table.** Item-level descriptive statistics (mean and standard deviation) for the Therapeutic Communication Scale in Nursing Students. (n=468) | | |  |
| --- | --- | --- | --- |
| Item | Mean | Standard Deviation (SD) | |
| I verbalize about clients’ situation or how they feel. | 3.33 | .667 | |
| I express how I understand the client by gestures and eye contact. | 3.58 | .664 | |
| I build a comfortable atmosphere for conversation. | 3.59 | .657 | |
| I recheck clients’ words and behaviors when they are not clear. | 3.37 | .642 | |
| I use an appropriate tone and volume when I talk. | 3.63 | .632 | |
| I give information easy enough for the client to understand. | 3.49 | .619 | |
| I notice changes in body language, facial expressions, and emotions even if the patient does not speak. | 3.32 | .723 | |
| I meet clients without prejudice. | 3.29 | .742 | |
| I explain I can’t approve unreasonable demands of the client. | 3.03 | .709 | |
| I don’t give advice or recommendations without clients’ approval. | 2.87 | .818 | |
| I don’t assume what the client will say. | 3.09 | .718 | |
| I only ask the patient one question at a time. | 3.03 | .767 | |
| I listen to the patient’s speech until the end without interrupting or blocking. | 3.35 | .700 | |
| I don’t reduce or exaggerate the clients’ problem. | 3.38 | .727 | |
| I give enough time for the client to organize their thoughts. | 3.35 | .672 | |

| **S1.2 Table**. Item-total statistics and Cronbach’s alpha if item deleted for the Therapeutic Communication Scale in Nursing Students. | | | | | |
| --- | --- | --- | --- | --- | --- |
|  | Scale Mean if Item Deleted | Scale Variance if Item Deleted | Corrected Item-Total Correlation | Squared Multiple Correlation | Cronbach’s Alpha if Item Deleted |
| I verbalize about clients’ situation or how they feel. | 46.37 | 36.100 | .558 | .336 | .872 |
| I express how I understand the client by gestures and eye contact. | 46.12 | 35.694 | .615 | .439 | .869 |
| I build a comfortable atmosphere for conversation. | 46.11 | 35.851 | .603 | .420 | .870 |
| I recheck clients’ words and behaviors when they are not clear. | 46.33 | 36.899 | .475 | .275 | .875 |
| I use an appropriate tone and volume when I talk. | 46.07 | 36.216 | .579 | .421 | .871 |
| I give information easy enough for the client to understand. | 46.21 | 36.271 | .586 | .377 | .871 |
| I notice changes in body language, facial expressions, and emotions even if the patient does not speak. | 46.38 | 36.249 | .488 | .304 | .875 |
| I meet clients without prejudice. | 46.41 | 35.741 | .533 | .337 | .873 |
| I explain I can’t approve unreasonable demands of the client. | 46.67 | 36.868 | .424 | .213 | .877 |
| I don’t give advice or recommendations without clients’ approval. | 46.83 | 36.486 | .391 | .224 | .880 |
| I don’t assume what the client will say. | 46.61 | 35.677 | .563 | .382 | .871 |
| I only ask the patient one question at a time. | 46.67 | 36.213 | .457 | .263 | .876 |
| I listen to the patient’s speech until the end without interrupting or blocking. | 46.35 | 35.752 | .571 | .402 | .871 |
| I don’t reduce or exaggerate the clients’ problem. | 46.31 | 35.008 | .637 | .448 | .868 |
| I give enough time for the client to organize their thoughts. | 46.34 | 35.571 | .623 | .445 | .869 |

**S1.3 Table.** Factor loadings of the Therapeutic Communication Scale in Nursing Students.

|  | **Factor 1: Relation building** | **Factor 2: Problem solving** |
| --- | --- | --- |
| I verbalize about clients’ situation or how they feel. | .616 |  |
| I express how I understand the client by gestures and eye contact. | .697 |  |
| I build a comfortable atmosphere for conversation. | .669 |  |
| I recheck clients’ words and behaviors when they are not clear. | .533 |  |
| I use an appropriate tone and volume when I talk. | .668 |  |
| I give information easy enough for the client to understand. | .643 |  |
| I notice changes in body language, facial expressions, and emotions even if the patient does not speak. | .563 |  |
| I meet clients without prejudice. | .537 |  |
| I explain I can’t approve unreasonable demands of the client. | .452 |  |
| I don’t give advice or recommendations without clients’ approval. |  | .424 |
| I don’t assume what the client will say. |  | .612 |
| I only ask the patient one question at a time. |  | .528 |
| I listen to the patient’s speech until the end without interrupting or blocking. |  | .669 |
| I don’t reduce or exaggerate the clients’ problem. |  | .727 |
| I give enough time for the client to organize their thoughts. |  | .714 |
